# Setting up a molecular diagnostic laboratory for SARS-CoV-2 testing: Experience of a single centre in a resource-constrained setting

**DOI:** 10.4102/ajlm.v10i1.1326

**Published:** 2021-03-30

**Authors:** Iriagbonse I. Osaigbovo, Isaac O. Igbarumah, Ekene B. Muoebonam, Darlington E. Obaseki

**Affiliations:** 1 Department of Medical Microbiology, School of Medicine, College of Medical Sciences, University of Benin, Benin City, Nigeria; 2 Department of Medical Microbiology, University of Benin Teaching Hospital, Benin City, Nigeria; 3 Molecular Virology Laboratory, University of Benin Teaching Hospital, Benin City, Nigeria; 4 Institute of Lassa Fever Research and Control, Irrua Specialist Teaching Hospital, Irrua, Nigeria; 5 Department of Anatomic Pathology, School of Medicine, College of Medical Sciences, University of Benin, Benin City, Nigeria; 6 Department of Anatomic Pathology, University of Benin Teaching Hospital, Benin City, Nigeria

**Keywords:** COVID-19, coronavirus disease 2019, laboratory, Nigeria, molecular diagnosis, SARS-CoV-2, severe acute respiratory syndrome coronavirus 2, UBTH, University of Benin Teaching Hospital, resource-constrained

## Abstract

**Background:**

Molecular detection of severe acute respiratory syndrome coronavirus 2 (SARS-CoV-2) is at the forefront of the global response to the coronavirus disease 2019 (COVID-19) pandemic. However, molecular diagnostic capabilities are poorly developed in many African countries. Efforts by the Nigeria Centre for Disease Control and other public health agencies to scale up facilities for molecular testing across the continent are well documented, but there are few accounts from the laboratories at the frontline.

**Intervention:**

As part of an institutional response to the COVID-19 pandemic, the University of Benin Teaching Hospital, Benin City, Nigeria, signed a memorandum of understanding with a World Bank-supported institution to obtain a non-proprietary testing platform, renovated an existing molecular virology laboratory and validated the test process to make SARS-CoV-2 testing readily available for decision-making by frontline health workers. These efforts resulted in the University of Benin Teaching Hospital’s inclusion in the Nigeria Centre for Disease Control COVID-19 molecular laboratory network. The laboratory achieved a turnover of 12 123 tests within 7 months of operation. Challenges faced and dealt with include incompatible equipment, limited skilled manpower, unstable (unreliable) electric power supply, disrupted procurement and supply chain, and significant overhead costs.

**Lessons learnt:**

Molecular diagnostic capability is essential in laboratory preparedness for pandemic response and can be achieved by establishing collaborative networks in low-resource settings.

**Recommendations:**

Molecular diagnostic capabilities attained during the COVID-19 pandemic should be maintained by governmental support of the local biotechnology sector, collaboration with partners and stakeholders and the expansion of diagnostics to include other diseases of public health importance.

## Background

Coronavirus disease 2019 (COVID-19) caused by severe acute respiratory syndrome coronavirus 2 (SARS-CoV-2) was first described following an outbreak of atypical pneumonia in China in late 2019.^1^ It has since escalated into a global pandemic with over 36 million cases and more than a million deaths worldwide as of 10 October 2020.^2^ Laboratory testing to detect, isolate and treat cases is key to the containment of the virus; establishing widespread diagnostic capacity has been a strategy of successful response in countries like South Korea.^3^ To date, molecular methods, particularly real-time reverse transcriptase polymerase chain reaction (PCR), remain the mainstay of diagnostic testing.^4^

**FIGURE 1:**
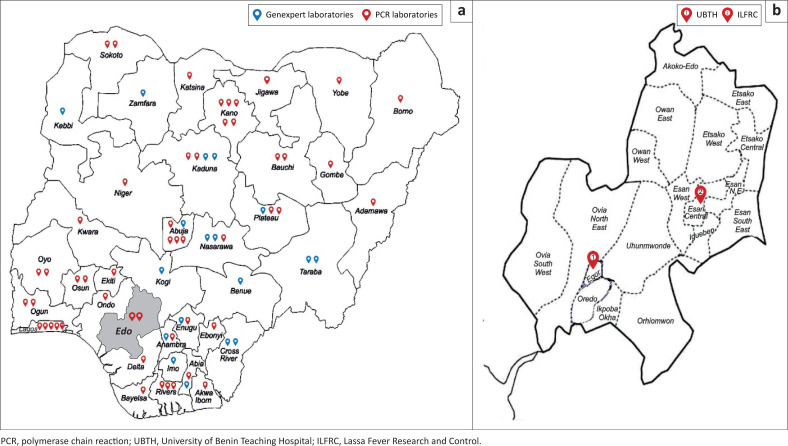
COVID-19 laboratory network in Nigeria and Edo state, 2020. (a) Map of Nigeria showing Nigeria Centre for Disease Control COVID-19 molecular laboratory network (public laboratories only). (b) Map of Edo state showing local government areas and the locations of University of Benin Teaching Hospital molecular virology laboratory and the Institute of Lassa Fever Research and Control, Irrua Specialist Teaching Hospital.

Molecular diagnostic capability is in short supply in many parts of sub-Saharan Africa. In Nigeria, available molecular diagnostic capabilities arose from vertical disease programme models for HIV and Lassa fever in the 2000s.^5,6,7^ Since the 2014 West African Ebola outbreak, which emphasised the need for strong public health systems, the Nigeria Centre for Disease Control (NCDC) has gradually developed a network of laboratories capable of molecular diagnosis of infections of public health importance.^8,9^ This enabled Nigeria to establish diagnostic capacity for SARS-CoV-2 testing in three laboratories within one month of the report of COVID-19 cases from China and before the detection of the first case in sub-Saharan Africa on 27 February 2020.^10,11^ By leveraging already existing infrastructure for HIV, Lassa fever and drug-resistant tuberculosis, the number of public and private laboratories able to test for SARS-CoV-2 has been gradually and consistently scaled up to 90 laboratories distributed across all 36 states and the federal capital territory of Nigeria as of 08 October 2020 (Figure 1a). The tremendous efforts by NCDC and other public health agencies across Africa to scale up testing are well documented in the scientific literature.^11^ It is desirable that laboratory testing for SARS-CoV-2 is made available close to the ‘frontline’ of the pandemic, that is, within hospitals whenever feasible and safe, as this enables clinical teams to make critical decisions about inpatient management.^12^ These decisions include whether to isolate patients, thereby preventing the nosocomial spread of COVID-19, and how to allocate scarce personal protective equipment (PPE). However, there is a paucity of accounts from laboratories at the frontline of testing and diagnosis.

Two public laboratories in the NCDC network for COVID-19 testing are domiciled in Edo state, Nigeria (Figure 1b). One of these, the Institute of Lassa Fever Research and Control (ILFRC), Irrua Specialist Teaching Hospital, is a pioneer laboratory conscripted early in the fight against COVID-19. The other, located at the University of Benin Teaching Hospital (UBTH) and included in the network on 10 May 2020, was set up because the management envisioned frontline diagnostic testing as a critical aspect of institutional response to the pandemic. This article describes the steps taken to set up testing for SARS-CoV-2 in the UBTH.

### Site description

The UBTH is an 850-bed tertiary hospital located in the South-South geopolitical zone of Nigeria. It serves Edo state which has a landmass of 17 802 square kilometres and neighbouring states Delta, Kogi, Anambra and Ondo, providing both primary and referral heathcare services. Located in the state capital Benin City, the institution is at the forefront of the state’s response to combating the pandemic.^13^ As of 31 December 2020, 2870 cases of laboratory-confirmed COVID-19 have been reported in Edo state.^14^

The molecular virology laboratory in UBTH is a biosafety level 2 containment facility. It was set up in 2005 and moved to its current location in 2011 with the support of the Institute of Human Virology of Nigeria to offer early infant diagnosis and viral load detection services for the United States President’s Emergency Plan for AIDS Relief AIDS Care Treatment in Nigeria programme in the state.^5^ This partnership provided training in PCR diagnostic testing for laboratory scientists as well as laboratory equipment including a conventional PCR machine (later replaced with the Roche COBAS® TaqMan 96, a proprietary real-time PCR machine not compatible with currently accessible SARS-CoV-2 assays). Other important equipment and items that were already in place in the laboratory include biosafety cabinets, fixed angle micro-centrifuge, bench centrifuge, heating blocks, freezers, an uninterrupted power supply and back-up generator. Besides HIV testing, the laboratory also recently joined the NCDC network for yellow fever and measles testing following a favourable performance in a laboratory audit exercise conducted in December 2019. The staff of six consists of three laboratory scientists, one laboratory technician and two data clerks.

## Description of the intervention

### Ethical considerations

This article followed all ethical standards for research without direct contact with human or animal subjects.

### Planning and preparation

#### Memorandum of understanding and polymerase chain reaction machine setup

**FIGURE 2:**
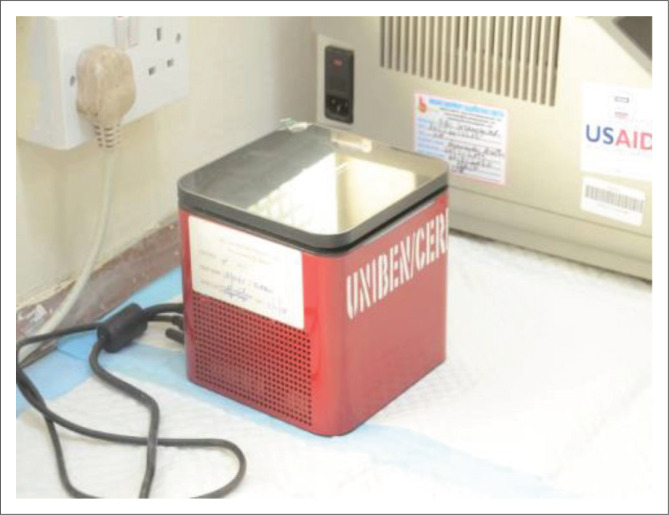
Magnetic induction cycler real-time Dx48 PCR instrument obtained from Centre for Excellence in Reproductive Health Innovation, Edo state, Nigeria, 2020.

To expedite the commencement of testing while sourcing funds to purchase a new PCR machine, the hospital collaborated with the Centre for Excellence in Reproductive Health Innovation, University of Benin, Nigeria. The Centre for Excellence in Reproductive Health Innovation is one of the several World Bank-funded centres for excellence in African higher education institutions initiated to promote homegrown and regional research networks. The collaboration with Centre for Excellence in Reproductive Health Innovation was made official by signing a memorandum of understanding on 15 April 2020. This memorandum of understanding clearly states the purpose of the agreement, which is to jointly operate a COVID-19 testing unit in UBTH. The areas and scope of cooperation (including equipment, personnel and biosafety, provision of consumables and data collection for research purposes), implementation and the mode of handling intellectual property that may arise from the collaboration were also spelt out. Following the signing of the memorandum of understanding, a new magnetic induction cycler real-time Dx48 PCR instrument (Biomolecular Systems, Upper Coomera, Queensland, Australia) belonging to the Centre for Excellence in Reproductive Health Innovation was transferred to the UBTH molecular laboratory to conduct SARS-CoV-2 testing. The machine is an open (non-proprietary) platform compatible for use with many assays (Figure 2).

#### Renovation of existing laboratory physical infrastructure

**FIGURE 3:**
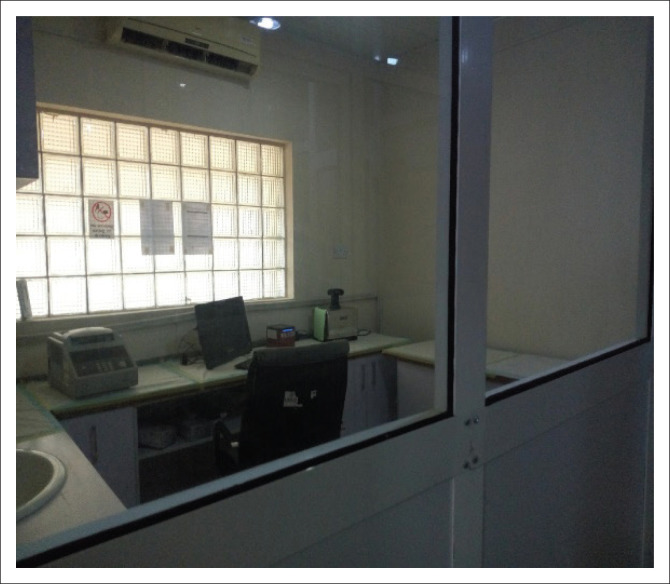
Amplification and detection room for real-time detection of nucleic acid, Edo state, Nigeria, 2020.

The existing laboratory was ill-designed for molecular testing of highly infectious diseases like SARS-CoV-2. Clean and dirty areas were not demarcated, that is, staff had to take the specimens through the master mix preparation area to get to the extraction room. The laboratory was thus physically reconfigured and renovated to demarcate the dirty (red) zone from clean areas to comply with a strictly unidirectional flow of movement. The cleanroom or section for mastermix preparation room, extraction room with a biosafety cabinet and centrifuge, and amplification and detection room for real-time detection of nucleic acid (Figure 3) were each equipped with a supply of PPE, multi-channel pipette and other equipment and consumables to prevent contamination. For increased biosafety, an exit room for doffing PPE and showering was created to ensure a strictly unidirectional movement and prevent contamination of administrative and office areas. A well-demarcated sample reception area was also created.

#### Online mentorship and training

To gain proficiency in the use of the machine, the chief scientist, other laboratory scientists and staff in the laboratory underwent hands-on training which, due to the lock-down situation in many states and ban on interstate travel in Nigeria, was done via online video conferencing. An experienced molecular biologist, conversant with the magnetic induction cycler testing platform and SARS-CoV-2 molecular testing provided the training. Sessions included the development of standard operating protocols and job aids specific for SARS-CoV-2 testing, biosafety protocols applicable to the virus and appropriate waste disposal.

#### Manpower reinforcement

Staff strength was reinforced by redeploying two scientists from the general medical microbiology laboratory and two data clerks. This scaled up the number of staff to 10: five laboratory scientists, one laboratory technician and four data clerks. The new staff received orientation and training for their specific tasks. Specifically, the laboratory technician is responsible for specimen reception and registration, the laboratory scientists carry out the various stages of reverse transcription-PCR and run quality control checks, while the data clerks are responsible for uploading data from the case investigation forms and test results onto online surveillance platforms.

#### Stockpiling of personal protective equipment

Personal protective equipment items and stock were increased to ensure adequate protection of laboratory staff. Personal protective equipment items included gloves, gowns, goggles, footwear, face shields, face masks and respirators.

### Evaluation and validation by Nigeria Centre for Disease Control

#### Site visit by officials of Institute of Lassa Fever Research and Control

As a regional reference laboratory for COVID-19 testing, ILFRC officials represented NCDC for the laboratory assessment visit using a checklist designed by the NCDC. Areas of assessment included biosafety checks, availability of PPE, a waste management plan, staff strength, equipment and stock management.

#### On-site mentorship, training and evaluation

An experienced molecular scientist from the ILFRC was deployed to provide technical support to the laboratory staff in workflow optimisation, infection prevention and control and handling of laboratory data. The UBTH laboratory scientists were also evaluated for competence in conducting the test using the national algorithm, laboratory safety, environmental cleaning and decontamination protocols.

#### Validation

Equipment validation was done using commercial standards and controls (Liferiver, Shanghai ZJ Biotech, Shanghai, China), while assay runs were validated by inter-laboratory comparison with ILFRC. Duplicate samples were collected from all suspected cases in the facility: one sample was sent to ILFRC while the other was processed and analysed by the on-site laboratory. Inter-laboratory concordance was measured and independent testing was commenced when 100% concordance was achieved in 40 samples.

### Operations

**FIGURE 4:**
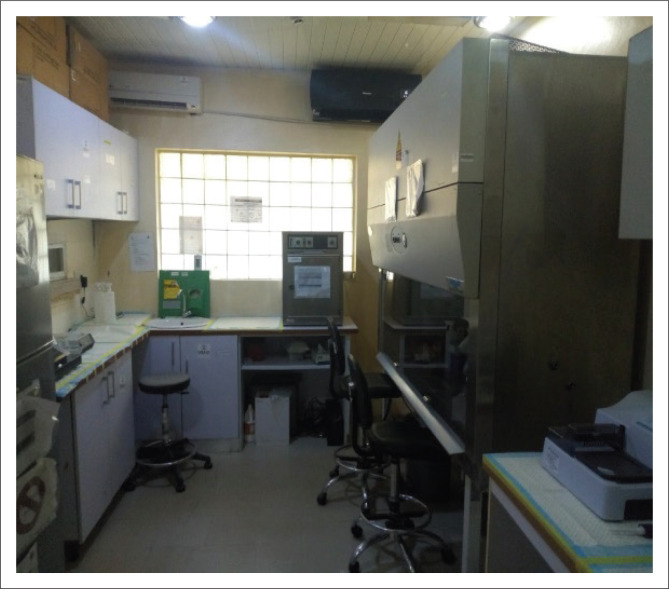
Sample inactivation room with a biosafety cabinet, Edo state, Nigeria, 2020.

Following a successful validation process and on the recommendation of the site evaluators from ILFRC, the UBTH molecular laboratory was included in the NCDC SARS-CoV-2 testing network on 10 May 2020. The testing protocol begins with inactivation of each sample, typically a nasopharyngeal swab and oropharyngeal swab in the same tube of viral transport medium, in a biosafety cabinet (Figure 4) using an external lysis buffer reagent, which accompanies the commercial RNA extraction kit. The sample is then moved to the RNA extraction room where RNA is extracted manually using an RNA isolation kit (Shanghai ZJ Biotech, Shanghai, China). Five microlitres (*μ*L) of the extracted RNA is then added to 20 *μ*L of prepared master mix (composed of 19 *μ*L supermix and 1 *μ*L enzyme mix) in the reaction tube. Polymerase chain reaction amplification was achieved using a novel coronavirus real-time multiplex reverse transcription-PCR kit (Liferiver, Shanghai ZJ Biotech, Shanghai, China) on a magnetic induction cycler real-time Dx48 PCR instrument (Biomolecular Systems, Upper Coomera, Queensland, Australia). Results were interpreted based on the detection of the envelope (E), nucleocapsid (N) and open reading frame 1ab (ORF1ab) genes. For a test to be considered positive, at least two of these genes, which must include the ORF1ab, must have been detected at the recommended cycle threshold (Ct) of less than 41.

Due to the open nature of the test platform, we were able to use other commercial RNA extraction and detection kits, for instance DAAN Gene (Da An Gene Co. Limited of Sun Yat-sen University, Guangzhou, China) was used when the Liferiver kit (Liferiver, Shanghai ZJ Biotech, Shanghai, China) was not available.

The real-time outbreak and epidemic surveillance software Surveillance Outbreak Response Management and Analysis System, employed by NCDC, was installed in the laboratory information management system and data clerks were trained by NCDC officials on test data imputation.

Quality assurance was emphasised along the entire testing pathway. Pre-analytical conditions were addressed by a clinical pathologist closely liaising with clinical staff on proper specimen collection, handling and transport. Quality control was ensured in each run by the use of appropriate positive, negative and internal controls. Equally, data were collected and analysed to monitor key performance indicators such as turnaround time, positivity and specimen rejection rates.

### Results

In seven months of operation, the laboratory has performed 12 123 assays on UBTH patient samples, referral samples from other health facilities and community samples from the Edo state government-driven community screening and testing efforts. Before the inclusion of the laboratory into the COVID-19 testing network, all samples from UBTH had to be sent in a once-daily trip to ILFRC which is over 100 km away and the result turnaround time was 3–4 days. The availability of on-site SARS-CoV-2 testing has shortened the turnaround time to 1–2 days as sample transportation-related delays in test turnaround time have been eliminated. The shortened sample collection-to-test result turnaround time expedites crucial patient care decision-making such as treatment initiation, triaging of patients in emergency departments, allocation of scarce PPE and other actions that prevent nosocomial spread. The renovations carried out in the laboratory improved the workflow and minimised the risks of contamination by ensuring that laboratory staff only move from clean areas to the dirty area (amplification area containing nucleic acids).

### Challenges

Establishing SARS-CoV-2 testing amid the COVID-19 pandemic has been problematic even in the most developed countries.^15,16^ General challenges include a slowdown in global manufacturing of sampling materials like swabs and viral transport medium, difficulties in shipping and procurement of commercial testing kits, contamination of molecular diagnostic reagents, lack of performance data for COVID-19 testing kits that have been approved for emergency use, lack of positive control materials for COVID-19 testing and shortage of PPE, among others. These obstacles are, expectedly, more pronounced in resource-poor settings, which also have their unique challenges.^17^ Currently, SARS-CoV-2 testing in Nigerian public health facilities is offered at no cost to individual patients and clients. Although reagents and consumables are provided by NCDC, the supply is often irregular and testing institutions bear the brunt of significant overhead running costs. To mitigate the impact of delays in supply of testing reagents, ample lead time is given in making requests for reagents to prevent stock-outs. In addition, the dedicated 30 kilovolt-amperes (kVa) back-up generator had to be replaced with a 60 kVa to cope with the unstable electric power supply to prevent disruptions in testing and ensure proper storage of samples (Table 1).

**TABLE 1:** Challenges of setting up of SARS-CoV-2 testing in University of Benin Teaching Hospital and mitigating actions taken, Edo state, Nigeria, 2020.

Challenge	Scope	Mitigating actions
Equipment incompatibility	The foremost challenge in joining the NCDC testing network was the lack of a compatible machine to do the test.	Collaboration with a World Bank-funded centre of excellence, Centre for Excellence in Reproductive Health Innovation, to acquire an open (non-proprietary) molecular testing platform.
Limited skilled manpower	Few laboratory personnel are conversant with polymerase chain reaction.	More scientists were cross-trained as expediently as possible to cushion the surge in testing for SARS-CoV-2.
Unstable and unreliable electric power supply	Molecular laboratories must have a constant supply of power to prevent disruptions in testing and provide proper storage for samples but this is inconsistent in Nigeria.	The dedicated 30 kVa back-up generator was replaced with a 60 kVa model to cope with increased utility. Solar panel inverters purchased prior to the renovation are also in use.
Procurement and supply chain disruptions	NCDC is responsible for the supply of sampling materials including viral transport medium, test reagents and consumables. There are sometimes delays in the supply of these materials brought about by factors such as shortages, transport and competitive demand.	Normal saline was used as a substitute viral transport medium during stock-outs. Ample lead time was employed in making requests for reagents to prevent stock-outs.
Sample volume-throughput mismatch	Due to the location of the laboratory in the capital city and a rollout of community testing by the state government, the sample volume received daily sometimes exceeds the capacity of the available machine which has a medium throughput. This has the potential to erode the advantage of shorter turnaround time for hospital samples.	Extra shifts were planned to expand throughput; hospital inpatient and emergency ward samples were given priority.
External quality assurance	For a long time, there was no established EQA programme for SARS-CoV-2 testing in Nigeria; however, at the time of this report, the NCDC recently set up an inter-laboratory comparison scheme in which samples from various laboratories are sent for retesting.	Validation of the testing process relied on inter-laboratory comparison with an established reference laboratory (Institute of Lassa Fever Research and Control).

EQA, external quality assurance; kVa, kilovolt-amperes; SARS-CoV-2, severe acute respiratory syndrome coronavirus 2; NCDC, Nigeria Centre for Disease Control.

## Lessons learnt

The experience of setting up the molecular laboratory during the COVID-19 pandemic emphasised the need for laboratory preparedness as a major aspect of institutional response to infectious disease outbreaks and other emergencies. Molecular diagnostic capability is key to this laboratory preparedness and can be achieved by establishing collaborative networks in low-resource settings. Firstly, UBTH was able to obtain a platform for testing by signing a collaborative memorandum of understanding with a funded research centre in the university. Secondly, despite travel restrictions, the laboratory staff were able to get the laboratory running via remote mentoring using web conferencing technology. The mentorship and guidance from the reference laboratory in the state was also invaluable particularly on the laboratory design, on-site evaluation and testing capacity validation.

Another lesson learnt is the value of non-proprietary molecular testing platforms that allow for flexibility and capacity to scale up in emergencies. This became especially evident when there were difficulties in accessing a particular commercial assay as the flexibility of the platform allowed us to easily switch to other available assay kits.

## Recommendations

The COVID-19 pandemic has demonstrated the essential role of diagnostics in the control of infectious diseases and sparked renewed interest in molecular technologies in low-resource settings, including Nigeria. We recommend that the capabilities attained are sustained by governmental support of the local biotechnology sector, continued surveillance for SARS-CoV-2 beyond the pandemic period, fostering collaboration and broadening the scope to include other diseases of public health importance. We expand on these recommendations below.

It is hoped that the rekindled interest in molecular diagnostics will bring about a burgeoning biotechnology sector including a network of biomedical engineering services and local sources for laboratory reagents and consumables. These are necessary for sustaining efforts at the institutional healthcare level. Indeed, this is already occurring in Nigeria; for instance, the viral transport medium required for the shipment of samples that was being imported at the start of the pandemic is now locally produced and supplied by the National Veterinary Research Institute in Vom, Jos. Laboratories with the capacity to develop and validate quality in-house assays should be supported to reduce the over-reliance on external commercial sources.

If SARS-CoV-2 becomes endemic in the population, surveillance for COVID-19 will need to be maintained. Low complexity, rapid point of care molecular testing platforms already in use in many hospitals can be leveraged to carry out smaller-scale testing and surveillance in the post-pandemic period when prevalence rates are likely to be lower. For instance, the Xpress SARS-CoV-2 test cartridge can be used in the GeneXpert® (Cepheid, Sunnyvale, California, United States) machine that is already in use for diagnosis of tuberculosis in many hospitals. Costs can be further lowered and throughput increased by deploying a pooled sample testing strategy after validating.^18^

There are no guarantees that government support for supplies of reagents, consumables and other logistics will last beyond the pandemic period. Sustaining operations in a molecular laboratory is capital intensive and it is pertinent to consider how this will be achieved in the future. As observed in several other contexts, capital investments in laboratory equipment are difficult to recoup especially where test volumes are low and overhead maintenance costs are high.^5,19^ Collaboration with an institution that had external funding was pivotal to the SARS-CoV-2 testing initiative in UBTH and collaboration will be what sustains capabilities attained going forward. Coordinated and goal-directed partnerships with national and regional stakeholders, research institutes, global donor agencies, international governing bodies and biomedical industries are required.^19^ Also, the trained workforce will need to be retained utilising incentives and new hands will need to be recruited.

While maintaining surveillance for SARS-CoV-2, laboratories, including UBTH, will need to broaden their focus to include other diseases of public health importance that can attract collaboration from both NCDC and external donor agencies invested in global health. Lassa fever, a disease endemic to many parts of Nigeria, is an attractive candidate. The World Health Organization also prescribes molecular diagnosis for some other relevant infectious diseases like sexually transmitted diseases and dengue fever in the essential diagnostics list currently in its second edition.^20^ Additionally, the COVID-19 outbreak has impressed the need to better define the epidemiology of severe acute respiratory illnesses of viral aetiology. These viruses are best detected by molecular means and it will be worthwhile attaining the tools necessary to differentiate them from the novel coronavirus.^21^

In conclusion, the successful setup of SARS-CoV-2 testing in UBTH was predicated on collaborative efforts, established quality management systems culture and innovativeness. Whatever the future focus of the molecular laboratory, it is pertinent that molecular diagnostic infrastructure is kept patent as part of both the laboratory and institutional preparedness plans. This will ensure swift mobilisation to deal with infectious disease crises that will almost inevitably arise in the future.
